# A cervical ligamentum flavum cyst in an 82-year-old woman presenting with spinal cord compression: a case report and review of the literature

**DOI:** 10.1186/1752-1947-6-92

**Published:** 2012-03-29

**Authors:** Alexandros G Brotis, Eftychia Z Kapsalaki, Evangelos K Papadopoulos, Kostas N Fountas

**Affiliations:** 1 Department of Neurosurgery, University Hospital of Larissa, School of Medicine, University of Thessaly, Larissa, Greece; 2 Diagnostic Radiology, University Hospital of Larissa, School of Medicine, University of Thessaly, Larissa, Greece

## Abstract

**Introduction:**

We report on a very rare case of a cervical ligamentum flavum cyst, which presented with progressive myelopathy and radiculopathy. The cyst was radically extirpated and our patient showed significant recovery. A review of the relevant literature yielded seven cases.

**Case presentation:**

An 82-year-old Greek woman presented with progressive bilateral weakness of her upper extremities and causalgia, cervical pain, episodes of upper extremity numbness and significant walking difficulties. Her neurological examination showed diffusely decreased motor strength in both her upper and lower extremities. Magnetic resonance imaging of her cervical spine demonstrated a large, well-demarcated cystic lesion on the dorsal aspect of her spinal cord at the C3 to C4 level, significantly compressing the spinal cord at this level, in close proximity to the yellow ligament and the C3 left lamina. The largest diameter of this lesion was 1.4 cm, and there was no lesion enhancement after the intravenous administration of a paramagnetic contrast. The lesion was surgically removed after a bilateral C3 laminectomy. The thick cystic wall was yellow and fibro-elastic in consistency, while its content was gelatinous and yellow-brownish. A postoperative cervical-spine magnetic resonance image was obtained before her discharge, demonstrating decompression of her spinal cord and dural expansion. Her six-month follow-up evaluation revealed complete resolution of her walking difficulties, improvement in the muscle strength of her arms (4+/5 in all the affected muscle groups), no causalgia and a significant decrease in her preoperative upper extremity numbness.

**Conclusion:**

Cervical ligamentum flavum cysts are rare benign lesions, which should be included in the list of differential diagnosis of spinal cystic lesions. They can be differentiated from other intracanalicular lesions by their hypointense appearance on T_1_-weighted and hyperintense appearance on T_2_-weighted magnetic resonance images, with contrast enhancement of the cystic wall. Surgical extirpation of the cyst is required for symptom alleviation and decompression of the spinal cord. The outcome of these cysts is excellent with no risk of recurrence.

## Introduction

It is well known, that cystic intracanalicular cervical spinal lesions may cause compression of the spinal cord and/or the exiting spinal nerve roots, resulting in the development of myelopathy and/or radicular symptomatology. This may eventually evolve to irreversible myelomalacia if the underlying pathology remains untreated. Various malignant pathological entities, as well as a large group of benign lesions, may present as solely or partially cystic lesions of the cervical spine. The malignant group includes cystic astrocytomas, schwannomas, meningiomas, ependymomas or metastatic lesions, while the benign lesion group includes synovial, ligamentum flavum, posterior longitudinal ligament, discal, perineural, arachnoid and dermoid cysts or hematomas [1]. These benign cysts usually accompany extensive degenerative changes and may worsen the chronic-standing symptoms and the neurological condition of the patient, delaying their accurate diagnosis and their proper management.

In our current communication, we report a case of a cervical ligamentum flavum cyst, presenting with severe progressive cervical myelopathy and radiculopathy. Our diagnostic and therapeutic approaches are presented, along with a systematic review of the pertinent literature.

## Case report

An 82-year-old Greek woman presented to the outpatient clinic of our department, complaining of bilateral weakness of her upper extremities and causalgia, as well as significant walking difficulties. Her symptoms started approximately three months earlier and were progressively worsening. Our patient reported that she had always had cervical pain and spontaneously resolving episodes of upper extremity numbness, but her current symptomatology was more intense and bothersome.

Her past medical history was significant for idiopathic arterial hypertension, diabetes mellitus, hypercholesterolemia, hypothyroidism, ischemic heart disease, depression and osteoporosis. Her past surgical history revealed surgically treated bilateral carpal tunnel syndrome several decades ago. Moreover, she had unilateral knee and hip arthroplasties three and 13 years ago respectively.

Her neurological examination showed diffusely decreased motor strength in both her upper and lower extremities. Her left arm muscle groups including deltoid, biceps and triceps showed 3/5 strength, while on her right side the same muscles showed 4/5 strength. Similarly, decreased muscle strength, mainly of her quadriceps and adductors, was noted in her lower extremities; 4/5 on her right and 3/5 on her left side. Burning pain and hypoesthesia were elicited upon examination in the distribution of the C4 and C5 sensory dermatomes, more prominent on her left side. There were no other light touch deficits. Her proprioception and temperature sensation were normal in both arms and legs. Her deep tendon reflexes were equally decreased in both biceps and triceps muscles bilaterally, while her right patellar reflex was less brisk than her left one. In addition, our patient complained of progressive gait impairment, which significantly limited her daily activities.

**Figure 1:**
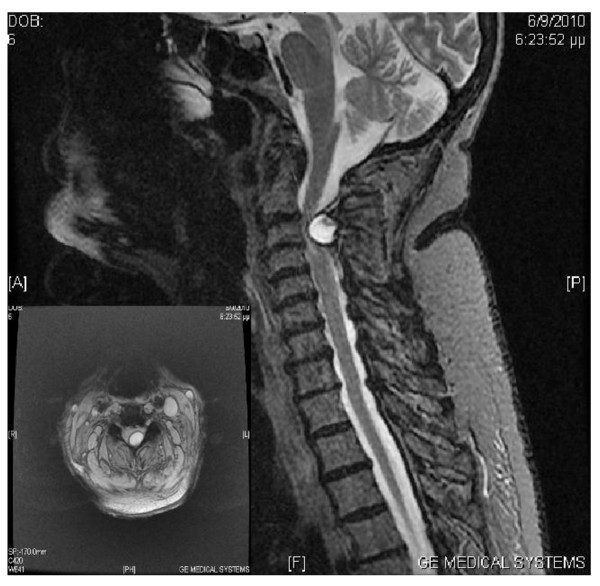
**Preoperative magnetic resonance imaging**. Mid-sagittal T2-weighted and axial T2-weighted images demonstrating an intracanalicular extradural space-occupying cystic lesion, which compresses and significantly displaces the spinal cord.

Magnetic resonance imaging (MRI) of her cervical spine demonstrated a large, well-demarcated cystic lesion on the dorsal aspect of her spinal cord at the C3 to C4 level (Figure 1). This lesion was significantly compressing her spinal cord at this level, and was in close proximity to the yellow ligament and the C3 left lamina. The largest diameter of this lesion was 1.4 cm, and there was no lesion enhancement after the intravenous administration of a paramagnetic contrast. Multilevel degenerative changes, including disc height decrease and dehydration, as well as multilevel facet hypertrophy were also noted on this study. The rest of her laboratory work up was unremarkable.

Under general endotracheal anesthesia and motor and somatosensory evoked potential intra-operative monitoring, our patient was positioned in the prone position, with her head secured on a three-point Mayfield fixation device.

**Figure 2:**
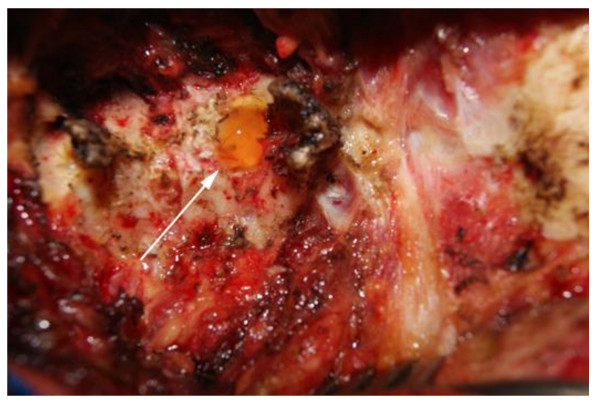
**Intra-operative photograph demonstrating the exposed ligamentum flavum cyst through the performed C3 laminectomy**.

A midline, vertical skin incision extending from her occiput to the C7 spinous process was made. Meticulous subperiosteal muscle dissection was performed and the spinal processes and the laminae of C2 to C4 were bilaterally exposed. The C3 spinous process was removed, and bilateral C3 laminectomies were performed. The C3 laminae had become paper-thin, most probably secondary to the long-standing pressure caused by the underlying cyst. A large, well-demarcated cyst was exposed, which occupied the epidural space and had displaced the underlying dura and spinal cord (Figure 2). The thick cystic wall was yellow and fibro-elastic in consistency, while its content was gelatinous and yellow-brownish. There was no connection between the cyst and the ipsilateral facet joint. The cyst was easily dissected from the dura and then was removed *in toto*, while the underlying displaced dura immediately expanded. Meticulous hemostasis was performed, and the surgical wound was closed in anatomical layers.

Our patient had an uneventful postoperative course. She was discharged on the third postoperative day, fully ambulating with significant improvement of her symptoms. Postoperative cervical spine MRI scans were obtained before her discharge, demonstrating decompression of her spinal cord and dural expansion. Her six-month follow-up evaluation revealed complete resolution of her walking difficulties, improvement of the muscle strength of both arms (4+/5 in all the affected muscle groups), no causalgia and a significant decrease in her preoperative upper extremity numbness.

The resected cyst had a firm, fibro-elastic wall, while its content was a yellowish thick gel. Microscopic examination demonstrated that the cystic wall had no lining, and there were no calcium or hemorrhagic deposits. Scattered histiocytes were observed in the cystic wall. No evidence of neovascularization was present in our specimen.

## Discussion

Intracanalicular extradural cysts represent a rare clinicopathological entity [2-8]. They present as space-occupying spinal canal lesions, usually in the lumbar and less frequently in the cervical area [5]. These cysts can be synovial, posterior longitudinal ligament, ligamentum flavum or discal, carrying distinct histological features and with different origins [5,6]. However, there is a quite confusing terminology in the literature, resulting in an ill-defined classification system [3,4,7-9]. Many authors have used the term 'synovial' cyst, for describing cysts originating from the facet joint, but also cysts originating from the ligamentum flavum, while others have used interchangeably the terms 'synovial' and 'ganglion' for describing cysts that were located in the extradural intracanalicular space [3,4,7-9]. In addition, a collective term 'juxtafacet' has also been used for describing synovial and ganglion cysts, which have different origins and histological characteristics [5,8].

Taking into consideration these terminology and classification issues, the incidence of true ligamentum flavum cysts is extremely low [2-9]. The majority of them occur in the lumbar region, while a total of seven cases of cervical ligamentum flavum cysts have been reported in the literature, as far as we know [3,4,6-9] (Table 1). These ligamentum flavum cysts occur in elderly patients, in their sixth decade of life or later (mean age at presentation: 70.2 years) [3,4,6-9]. This may be related to the increased incidence of degenerative spinal changes, which seems to co-exist in all the reported cases. No evidence of sex predilection can be established from the analysis of the literature data, due to the very limited number of cases (six men and two women) [3,4,6-9]. Similarly, no racial predilection may be established, due to the limited number of cases, although the majority of the reported cases come from Japan [3,4,6-9]. It has been reported that ligamentum flavum cysts are usually located laterally and not in the anatomical midline, since the ligament is thicker and denser in the midline [5,9].

**Table 1:** Demographic characteristics, anatomic location and surgical management of previously reported cervical ligamentum flavum cyst cases.

Reference	Country of origin	Number of patients	Age (years), sex	Anatomic location	Concomitant spinal pathology	Surgical treatment
[7]	Japan	1	72, male	C3 to C4	C3 to C5 degenerative changes	Laminectomy
[8]	Japan	2	81, male	C3 to C4	C3 to C4 degenerative changes	Laminoplasty
			65, male	C3 to C4	C3 to C4 degenerative changes	Laminoplasty
[9]	France	1	73, male	C4 to C5	Degenerative changes	Laminectomy
[4]	Japan	1	66, male	C7 to T1	C5 to T1 degenerative changes	Laminoplasty
[6]	Japan	1	63, female	C4 to C5	Rheumatoid arthritis	Laminectomy and fusion
[3]	Italy	1	60, male	C4 to C5	Degenerative changes	Laminectomy
This case	Greece	1	82, female	C3 to C4	Degenerative changes	Laminectomy

The exact pathophysiologic mechanism responsible for ligamentum flavum cyst development remains unclear [2,5-8]. It has been postulated that repetitive mechanical trauma of the ligamentum flavum, caused by the chronically degenerated vertebral column, results in a progressive degeneration of its elastic component [3,5,6,9]. These changes, along with the replacement of its elastic with less flexible collagen fibers, induce small tears in the ligamentum flavum, which progressively undergo myxoid degeneration, regional necrosis and, finally, microcystic changes [3,5,6,9]. These microcysts progressively enlarge and may coalesce, thus producing a ligamentum flavum cyst [3,5,6,9]. The proposed pathophysiological mechanism is supported by the fact that the vast majority of ligamentum flavum cysts occur in patients with degenerative spinal changes, and also in relatively mobile areas of the cervical or lumbar spine, where mechanical trauma and osteophyte formation are more frequent [3,5,6,9]. Indeed, all the reported cases of cervical ligamentum flavum cysts occurred in patients older than 60 years, with co-existent marked degenerative cervical spine changes, and at relatively mobile intervertebral spaces [2-4,6-9].

The ligamentum flavum cysts may enlarge and can cause cervical spine stenosis, especially in patients with concomitant degenerative changes [2-4,6-9]. In our case, our patient became unable to walk due to her severe progressive myelopathy, secondary to spinal cord compression at the C3 level. However, in other cases when the cyst is eccentrically located, radiculopathy may be the most prominent symptom [4,5,7]. The clinical presentation of ligamentum flavum cysts may mimic other space-occupying spinal canal lesions. Their presentation, however, is more progressive and slower compared to other space-occupying lesions, such as tumors or hematomas.

MRI of the vertebral column is considered the method of choice for the diagnosis of patients with intracanalicular space-occupying lesions. Ligamentum flavum cysts appear as well-demarcated, isointense lesions on T1-weighted images, while they are usually hyperintense on T2-weighted images [4-9]. However, they may demonstrate hypointense periphery and hyperintense core on T2-weighted images [3]. There is usually enhancement of the cystic wall after the intravenous administration of a paramagnetic agent, although this is not always observed (as in our case) [3]. Their differential diagnosis includes meningiomas, schwannomas, metastases, fibrous dysplasia, neurofibromas, ependymal cysts, juxta-articular cysts, perineural cysts, infectious cysts, arachnoid cysts, dermoid cysts or rheumatoid pannus [1,5]. However, their smooth, well-defined margins, the absence of any infiltrative characteristics and their T1 and T2 magnetic resonance characteristics can accurately differentiate them from all other space-occupying intracanalicular cervical lesions. Myelogram and post-myelogram computed tomography had been previously used in the imaging of patients with possible ligamentum flavum cysts [7]. However, MRI provides a more accurate, more specific and a non-invasive preoperative evaluation in these patients.

Surgical extirpation of the cyst through bilateral or unilateral laminectomy, depending on their location and their size, constitutes the treatment of choice in all the reported cases [3,4,6-9]. It has to be emphasized that the cystic wall has to be dissected from the underlying dura and the surrounding ligamentum flavum, although dense adhesions may be present, as in our case, due to the long-standing mechanical pressure. The vast majority of the previously reported cases describe the presence of a well-defined plane between the cyst and the ligamentum flavum, which always allowed complete cyst resection [3,4,6-9]. In one of the reported cases, a fusion was performed along with the laminectomy, while in two other reports, laminoplasty was employed, apparently for stabilization purposes [4,6,8]. However, in all other cases, laminectomies, with no further instrumentation and fusion, were performed [3,7,9]. In our case, single level bilateral laminectomies were performed with no need for any stabilization. No cases of cyst recurrences have been described so far in the literature.

The macroscopic appearance of the ligamentum flavum cysts is that of a fibrous, thick cystic wall with gelatinous or mucinous yellowish content [2,5,6,8,9]. Calcium deposits may be found in the cystic wall [7,9]. Microscopically, there is no synovial lining, while ligamentum flavum myxoid and pseudocystic degeneration with chondrocytic metaplasia, fragmentation of the elastic fibers and hyalinization of the collagen fibers are usually present [5,6]. The adjacent ligamentum flavum may demonstrate inflammatory cell infiltrations and mild to moderate neovascularization [3,5,8]. Degenerative clefts, myxoid inclusions and/or hemorrhagic elements may occasionally be found in the cystic content [2,8].

Complete cyst removal is associated with complete or nearly complete symptom resolution and overall excellent clinical outcome. In the vast majority of the reported cases the preoperative symptoms were usually resolved within a period of a few weeks of the operation, as in our case [3,5,7-9]. Cyst recurrences have not been reported in cases of surgical extirpation [3,5-8]. However, in cases where it is not possible to completely resect the cystic wall due to the development of dense adhesions with the underlying dura, recurrence is possible [5]. Wildi *et al. *[10] have reported cyst recurrence in the lumbar region in cases of incomplete resection within the first postoperative year.

## Conclusions

The presence of a cystic mass on a cervical spine MRI study should always raise the possibility of a ligamentum flavum cyst, although these cysts represent a quite rare pathological entity. The MRI features that may differentiate them from other intracanalicular lesions are their hypointense appearance on T1 and hyperintense appearance on T2 imaging, with contrast enhancement of the cystic wall. Surgical extirpation of the cyst is required for symptom alleviation and decompression of the spinal cord. Spinal fusion usually is not necessary, unless wide exposure and multiple level laminectomies were required for a large cyst removal. The outcome of these cysts is excellent with no risk of recurrence.

## Consent

Written informed consent was obtained from the patient for publication of this case report and any accompanying images. A copy of the written consent is available for review by the Editor-in-Chief of this journal.

## Competing interests

The authors declare that they have no competing interests.

## Authors' contributions

All authors contributed in writing the manuscript. All authors read and approved the final manuscript.
